# Comparison of efficacy between anti-vascular endothelial growth factor (VEGF) and laser treatment in Type-1 and threshold retinopathy of prematurity (ROP)

**DOI:** 10.1186/s12886-018-0685-6

**Published:** 2018-01-30

**Authors:** Zijing Li, Yichi Zhang, Yunru Liao, Rui Zeng, Peng Zeng, Yuqing Lan

**Affiliations:** Department of Ophthalmology, Guangdong Provincial Key Laboratory of Malignant Tumor Epigenetics and Gene Regulation, Sun Yat-sen Memorial Hospital, Sun Yat-sen University, Guangzhou, People’s Republic of China

**Keywords:** ROP, Anti-VEGF, Laser, Treatment

## Abstract

**Background:**

Retinopathy of Prematurity (ROP) is one of the most common causes of childhood blindness worldwide. Comparisons of anti-VEGF and laser treatments in ROP are relatively lacking, and the data are scattered and limited. The objective of this meta-analysis is to compare the efficacy of both treatments in type-1 and threshold ROP.

**Methods:**

A comprehensive literature search on ROP treatment was conducted using PubMed and Embase up to March 2017 in all languages. Major evaluation indexes were extracted from the included studies by two authors. The fixed-effects and random-effects models were used to measure the pooled estimates. The test of heterogeneity was performed using the Q statistic.

**Results:**

Ten studies were included in this meta-analysis. Retreatment incidence was significantly increased for anti-VEGF (OR 2.52; 95% CI 1.37 to 4.66; *P* = 0.003) compared to the laser treatment, while the incidences of eye complications (OR 0.29; 95% CI 0.10 to 0.82; *P* = 0.02) and myopia were significantly decreased with anti-VEGF compared to the laser treatment. However, there was no difference in the recurrence incidence (OR 1.86; 95% CI 0.37 to 9.40; *P* = 0.45) and time between treatment and retreatment (WMD 7.54 weeks; 95% CI 2.00 to 17.08; *P* = 0.12).

**Conclusion:**

This meta-analysis indicates that laser treatment may be more efficacious than anti-VEGF treatment. However, the results of this meta-analysis also suggest that laser treatment may cause more eye complications and increase myopia. Large-scale prospective RCTs should be performed to assess the efficacy and safety of anti-VEGF versus laser treatment in the future.

**Electronic supplementary material:**

The online version of this article (10.1186/s12886-018-0685-6) contains supplementary material, which is available to authorized users.

## Background

Retinopathy of Prematurity (ROP) is one of the most common causes of childhood blindness worldwide [1]. ROP is a vasoproliferative disorder of the retina associated with preterm infants. In 2003, the Early Treatment for Retinopathy of Prematurity Cooperative Group (ETROP) suggested that type-1 and threshold ROP should be treated [2].

In the past few decades, laser photocoagulation has been frequently used to treat ROP [3–6]. The application of laser treatment remains controversial because of its side-effects, including visual field loss, high myopia and retinal destruction [7–9]. Although the pathogenesis of ROP is incompletely understood, vascular endothelial growth factor (VEGF) has been considered to be one of the key mechanisms in vasculogenesis and angiogenesis. In recent years, anti-VEGF treatment has been used in ROP [9–12]. The Bevacizumab Eliminates the Angiogenic Threat of Retinopathy of Prematurity study (BEAT-ROP) [13] showed that VEGF inhibitors have more satisfactory outcomes than laser photocoagulation in zone I ROP. However, the best way to treat ROP, especially type-1 and threshold ROP, is under debate due to the complications, refractive error and systemic side-effects of both treatments [14–19].

Comparisons of anti-VEGF and laser treatments in ROP are relatively lacking, and the data are scattered and limited. Thus, randomized controlled trials (RCTs) and comparative non-randomized studies (CNSs) that provide high-quality data are included in this meta-analysis. The objective of this meta-analysis is to compare the efficacy of both treatments in type-1 and threshold ROP, including recurrence incidence, retreatment incidence, eye complication incidence, spherical equivalent at the last follow-up visit and the time between treatment and retreatment.

## Methods

### Evidence acquisition

This meta-analysis is reported according to the Preferred Reporting Items for Systematic Reviews and Meta-analyses (PRISMA statement) [20].

### Data sources and literature search strategy

A comprehensive literature search was conducted in several databases from the earliest available dates to March 2017, in all languages. The databases included PubMed and Embase.

The terms “retinopathy of prematurity” or “ROP” were searched. The related-articles function was also applied to broaden the search. Relevant articles were manually searched in the reference lists of all studies.

### Study selection

After importing all retrieved articles into Endnote X4 (Thomson Corporation, America) and removing duplicates using the “Find Duplicates” function, screening of the identified studies was performed by two independent authors based on the titles and abstracts. Irrelevant studies were excluded, and full-text screening was performed for eligibility of final inclusion.

RCTs and CNSs that compared VEGF inhibitors with laser treatment and provided at least one of the quantitative outcomes (recurrence or retreatment incidence) were included. Studies that lacked comparisons and that selected patients without type-1 and threshold ROP were excluded. Comments, letters to the editor, editorials, case reports, conference abstracts, experimental animal studies and review articles were also excluded.

### Data extraction

Two authors extracted data separately for the following details in the anti-VEGF and laser treatment groups: first author, publication year, country, study design, single-centre or multi-centre study, follow-up time, whether informed consent was obtained, time between treatment and retreatment, sample size, recurrence number, retreatment number, eye complication number and spherical equivalent at the last follow-up. Recurrence was defined as any of the following: termination of development in retinal vascularization, development of a demarcation line with or without a plus sign (plus sign: tortuous and dilated vessels or iris neovascularization with/without vitreous opacities), recurrent neovascularization and haemorrhage, recurrent plus sign, or progression of traction despite treatment. Retreatment was defined as the following: treatments that were applied because of recurrence after the initial laser or anti-VEGF treatment. Eye complications, including corneal opacity, cataract, preretinal or intravitreal haemorrhage and retinal detachment, were recorded. Any disagreements or differences in the data were resolved by consensus of the senior authors.

### Methodological quality assessment

Two authors rated all of the included studies for the level of evidence according to criteria provided by the Center for Evidence-Based Medicine in Oxford [21]. The quality of the RCTs was evaluated using Cochrane risk of bias tool [22]. The quality of the CNSs was evaluated using the ROBINS-I assessment tool [23].

### Statistical analysis

Data extracted from the articles were analysed in different subgroups (RCTs and CNSs) using Review Manager V5.3.5 (Cochrane Collaboration, London, UK). Dichotomous and continuous variables were compared using the odds ratio (OR) and weighted mean difference (WMD), respectively. The fixed-effect model was applied, and heterogeneity was quantified using the I^2^ value. When the Cochrane Q-test *P* value was >0.1, heterogeneity was considered to be not statistically significant, and the random-effects model was used to address within-study and between-study variances. An I^2^ value that was less than 25%, between 25% and 50% and more than 50% was defined as low, moderate and high heterogeneity, respectively.

## Results

### Study selection

**Fig. 1:**
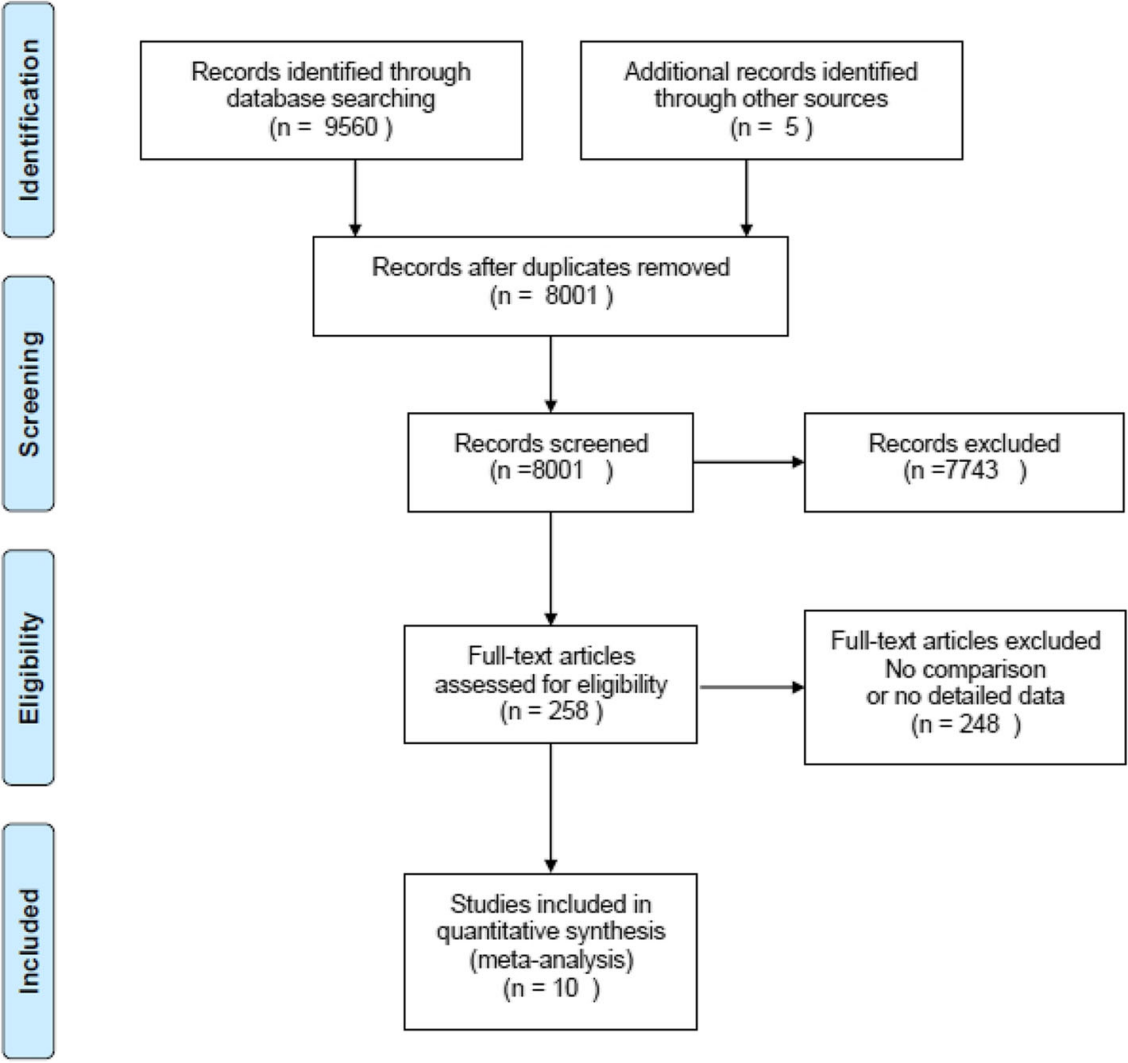
PRISMA flow diagram for study selection. Reprinted with permission From: Moher D, Liberati A, Tetzlaff J, Altman DG, The PRISMA Group (2009).Preferred Reporting Items for Systematic Reviews and Meta-Analyses: The PRISMA Statement. PLoS Med 6(7): e1000097. doi:10.1371/journal.pmed1000097

The flow diagram of the study selection is shown in Fig. 1. Nine thousand five hundred sixty-five records were identified from the database search and other sources. Eight thousand one records were screened after duplicates were removed. A total of 258 full-text articles were assessed for eligibility according to the title and abstract. Eventually 10 studies [7–9, 11–13, 16, 24–26] that had comparisons and provided detailed quantitative data were included in this meta-analysis.

### Study characteristics and methodological quality assessment

**Fig. 2:**
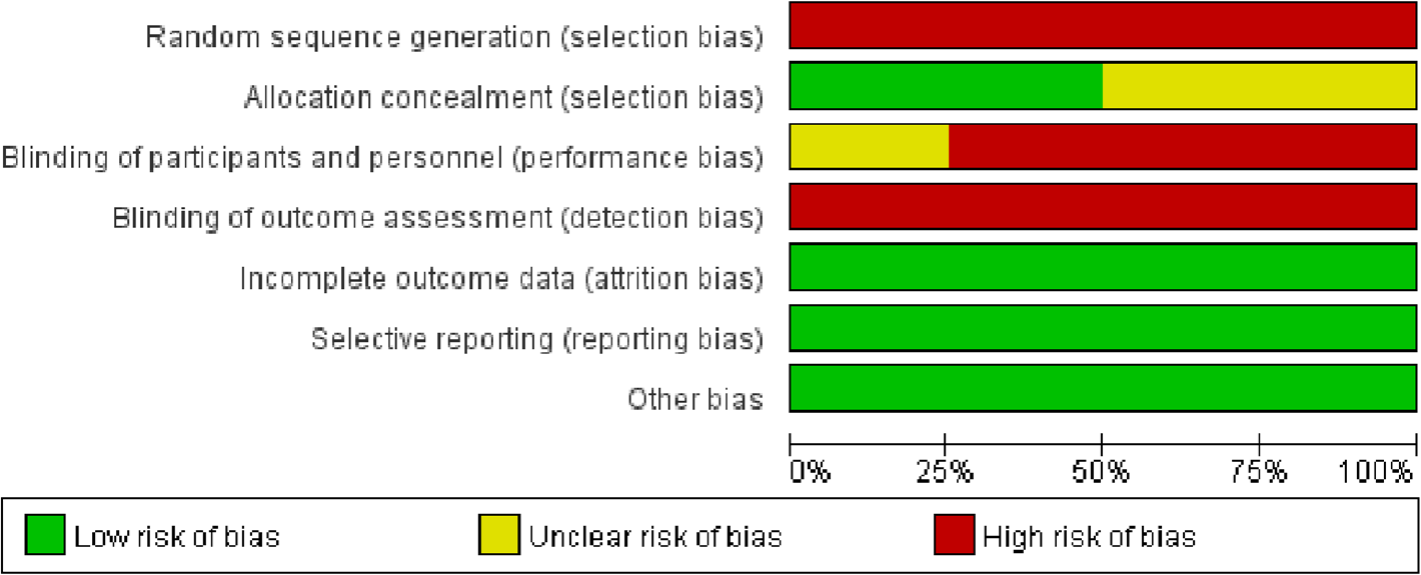
Quality assessment of randomized controlled trials

Characteristics of included studies are shown in Table 1. Four studies were RCTs [12, 13, 24, 25] (evidence level: 2b), and 6 were CNSs [7–9, 11, 16, 26] (evidence level: 3). Informed consent was obtained in all included studies. Three studies were multi-centre studies [11, 13, 26] and the rest [7–9, 12, 16, 24, 25] were single-centre studies. Each study had a follow-up time of no less than 6 months. However, the follow-up time was unclear in one study [26]. The quality assessment of 4 RCTs is shown in Table 2 and Fig. 2. The included RCTs had an overall medium risk of bias. All CNSs were judged to be at an overall moderate risk of bias according to the ROBINS-I assessment tool (shown in Table 3).

**Table 1:** Characteristics of the included studies

First Author & Year of Publication	Country	Study design	ICO	S/M	level of evidencea	VEGF Inhibitors						Laser						MFT(m)
						SS	Rec	Ret	Com	SE(D)	TTR(w)	SS	Rec	Ret	Com	SE(D)	TTR(w)	
Mintz-Hittner 2011 [13]	USA	RCT	YES	M	2b	140	6/4.3	NG	2/1.4	NG	NG	146	32/21.9	NG	6/4.1	NG	NG	8
Harder 2013 [16]	Germany	CNS	YES	S	3	23	0/0	0/0	0/0	−1.04 ± 4.24	NG	26	1/3.8	1/3.8	1/3.8	−4.41 ± 5.50	NG	12
Moran 2014 [12]	Ireland	RCT	YES	S	2b	14	3/21.4	3/21.4	NG	NG	16.00 ± 1.00	14	1/7.1	1/7.1	NG	NG	2.00 ± 0.01	24
Lepore 2014 [24]	Italy	RCT	YES	S	2b	12	0/0	0/0	0/0	NG	NG	12	1/8.3	1/8.3	1/8.3	NG	NG	9
Isaac 2015 [8]	Canada	CNS	YES	S	3	23	0/0	0/0	0/0	−3.57 ± 6.19	NG	22	1/4.5	1/4.5	0/0	−6.39 ± 4.41	NG	>9
Hwang 2015 [9]	USA	CNS	YES	S	3	22	3/13.6	NG	0/0	2.40 ± 3.50	9.00 ± 5.70	32	1/3.1	NG	6/18.8	−5.30 ± 5.40	2.60 ± 0.01	6–40
Gunay 2016 [11]	Turkey	CNS	YES	M	3	133	28/21.1	12/9.0	NG	NG	NG	111	1/0.9	0/0	NG	NG	NG	18
Karkhaneh 2016 [25]	Iran	RCT	YES	S	2b	86	9/10.5	9/10.5	0/0	NG	5.07 ± 1.66	72	1/1.4	1/1.4	0/0	NG	3 ± 0.01	22.5
Mueller 2016 [7]	Germany	CNS	YES	S	3	74	7/9.5	5/6.8	1/1.4	NG	NG	34	0/0	0/0	4/11.8	NG	NG	12
Walz 2016 [26]	Germany	CNS	YES	M	3	33	NG	5/15.1	NG	NG	NG	129	NG	18/14.0	NG	NG	NG	–
						560						598						

RCT, Randomized Controlled Trial; CNS, Comparative Non-randomized Study; ICO, Informed Consent Obtained; S/M, Single−/Multi-centre; SS, Sample Size (eye number); Rec, Recurrence number/incidence(eye number/incidence); Ret, Retreatment number/incidence (eye number/incidence); Com, Complication number/incidence (eye number/incidence); SE(D), Spherical Equivalent at Last Follow-up (Dioptre); TTR(w), Time between Treatment and Retreatment (week); MFT, Mean Follow-up time (months); NG, Not Given

Level of Evidence^a^: according to the criteria by the Center for Evidence-Based Medicine [21]

**Table 2:** Quality assessment of randomized controlled trials

Domain	Review authors’ judgement	Option	Mintz-Hittner 2011 [13]	Moran 2014 [12]	Lepore 2014 [24]	Karkhaneh 2016 [25]
Sequence generation	Was the allocation sequence adequately generated?	Yes/Unclear/No	YES	Unclear	YES	Unclear
Allocation concealment	Was allocation adequately concealed?	Yes/Unclear/No	NO	NO	NO	NO
Blinding of participants and personnel	Was knowledge of the allocated intervention adequately prevented during the study?	Yes/Unclear/No	NO	NO	Unclear	NO
Blinding of outcome assessors	Was knowledge of the allocated intervention adequately prevented during the study?	Yes/Unclear/No	NO	NO	NO	NO
Incomplete outcome data	Were incomplete outcome data adequately addressed?	Yes/Unclear/No	YES	YES	YES	YES
Selective outcome reporting	Are reports of the study free of suggestion of selective outcome reporting?	Yes/Unclear/No	YES	YES	YES	YES
Other sources of bias	Was the study apparently free of other problems that could put it at a high risk of bias?	Yes/Unclear/No	YES	YES	YES	YES

**Table 3:** Quality assessment of comparative non-randomized studies

Studies	Country	Pre-intervention and at-intervention domains			Post-intervention domains				Overall risk of bias
		Bias due to confounding	Bias in selection of participants in the study	Bias in classification of interventions	Bias due to deviations from intended interventions	Bias due to missing data	Bias in measurement of outcomes	Bias in selection of the reported result	
Harder 2013 [16]	Germany	M	L	L	L	L	M	L	M
Isaac 2015 [8]	Canada	M	M	L	L	L	M	L	M
Hwang 2015 [9]	USA	M	L	L	L	L	M	L	M
Gunay 2016 [11]	Turkey	M	M	M	L	L	M	L	M
Mueller2016 [7]	Germany	M	M	M	L	L	M	L	M
Walz 2016 [26]	Germany	M	L	L	L	M	L	L	M

L, low risk of bias; M, moderate risk of bias.

### Efficacy outcomes

**Fig. 3:**
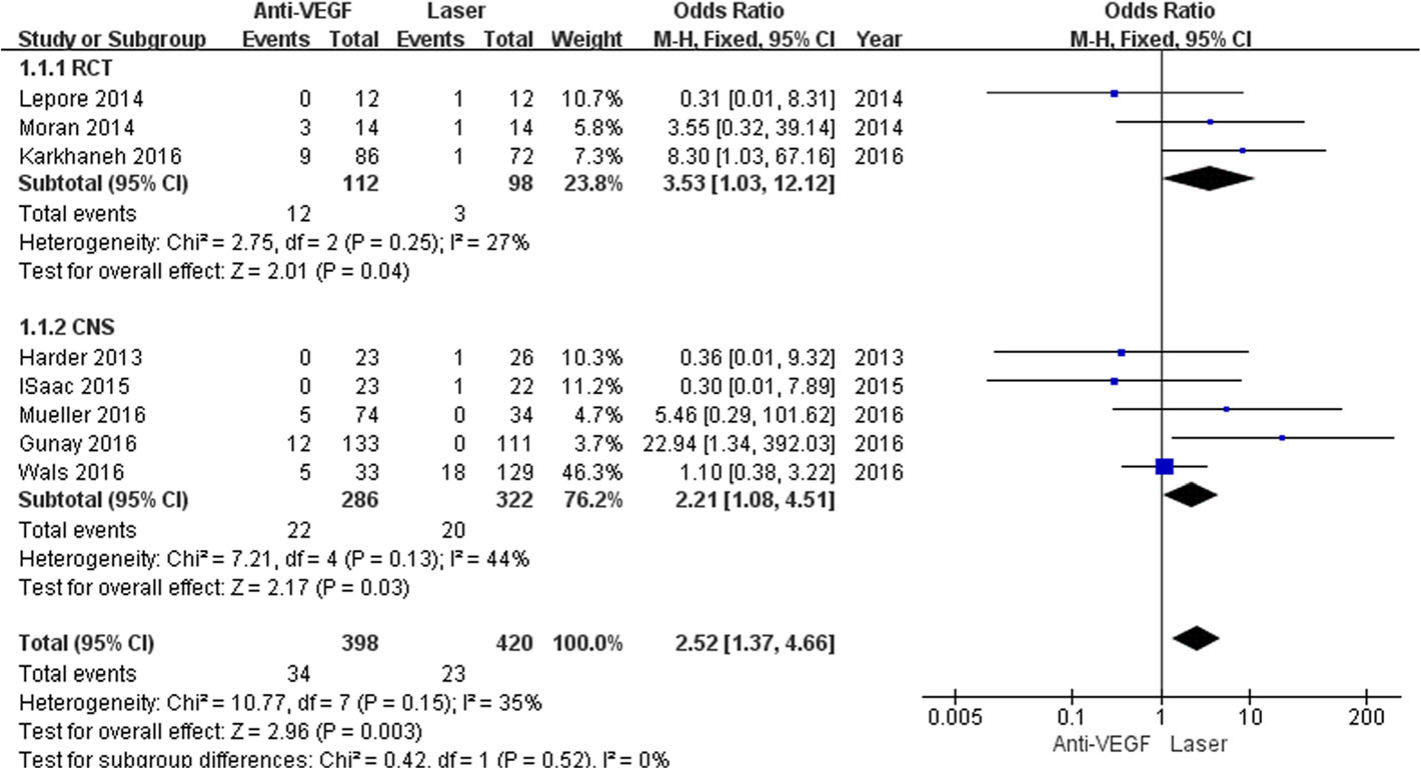
Forest plots depicting retreatment incidence reported in the included studies. ORs are shown with 95% CIs

**Fig. 4:**
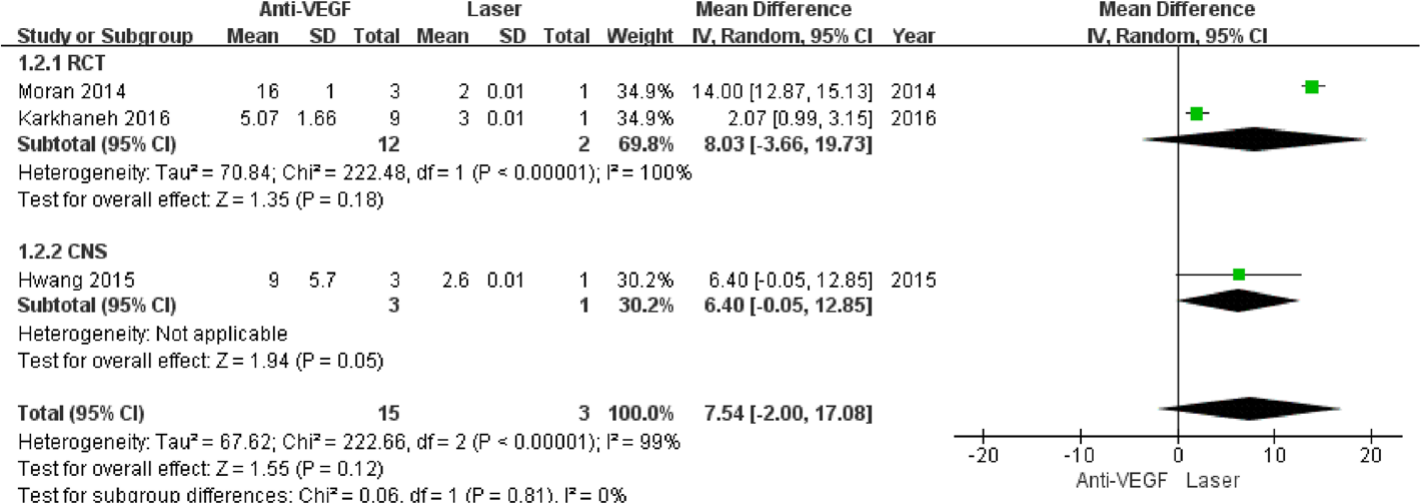
Forest plots depicting time between treatment and retreatment reported in the included studies. ORs are shown with 95% CIs

**Fig. 5:**
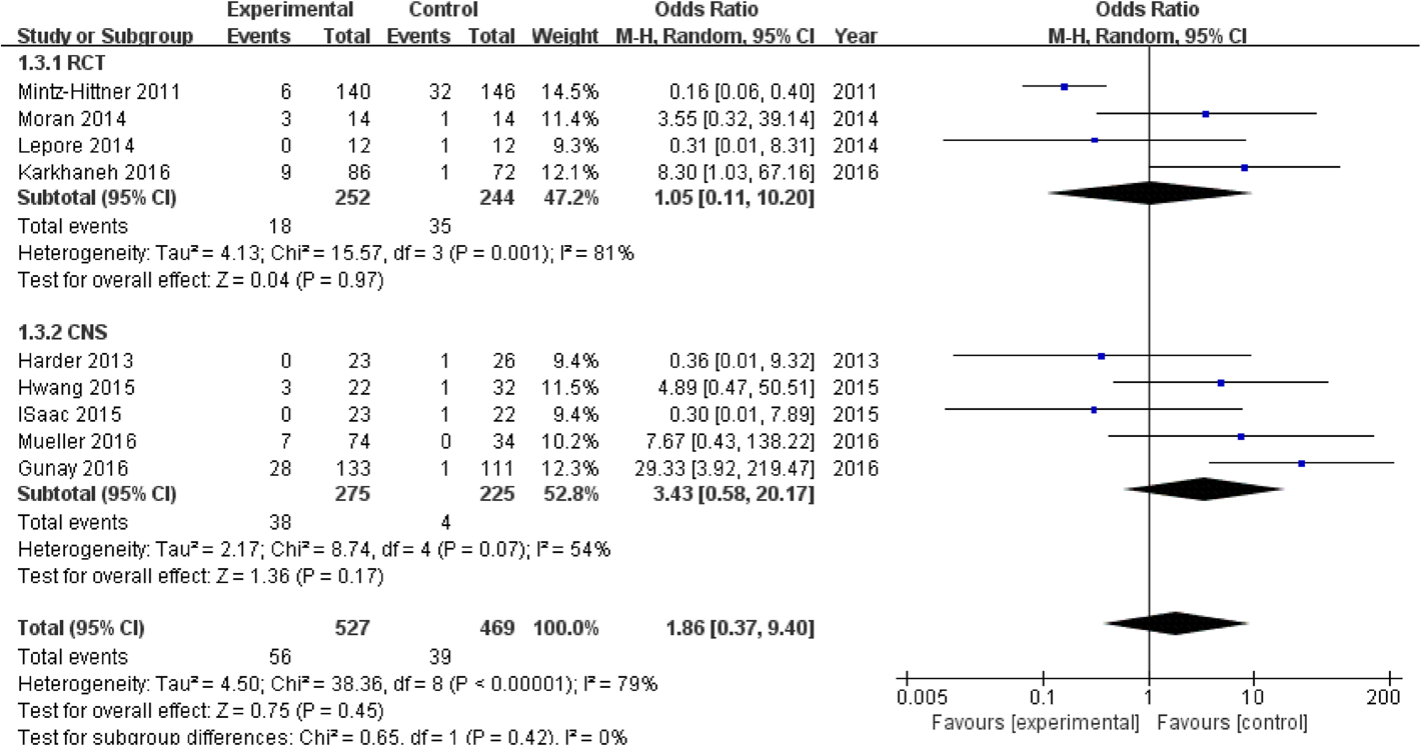
Forest plots depicting recurrence incidence reported in the included studies. ORs are shown with 95% CIs

In both subgroups, the retreatment incidence was significantly increased in anti-VEGF (RCT: OR 3.53, 95% CI 1.03 to 12.12, *P* = 0.04; CNS: OR 2.21, 95% CI 1.08 to 4.51, *P* = 0.03) compared to laser with low heterogeneity (RCT: I^2^ = 27%, *P* = 0.25; CNS: I^2^ = 44%, *P* = 0.13) (Fig. 3). There was no difference in terms of time between treatment and retreatment, and the WMDs were 7.54 weeks (95% CI 2.00 to 17.08; *P* = 0.12) between the groups (Fig. 4). The same result was observed in terms of recurrence incidence in both subgroups (RCT: OR 1.05, 95% CI 0.11 to 10.20, *P* = 0.97; CNS: OR 3.43, 95% CI 0.58 to 20.17, *P* = 0.17) (Fig. 5).

### Eye complication and refractive outcomes

**Fig. 6:**
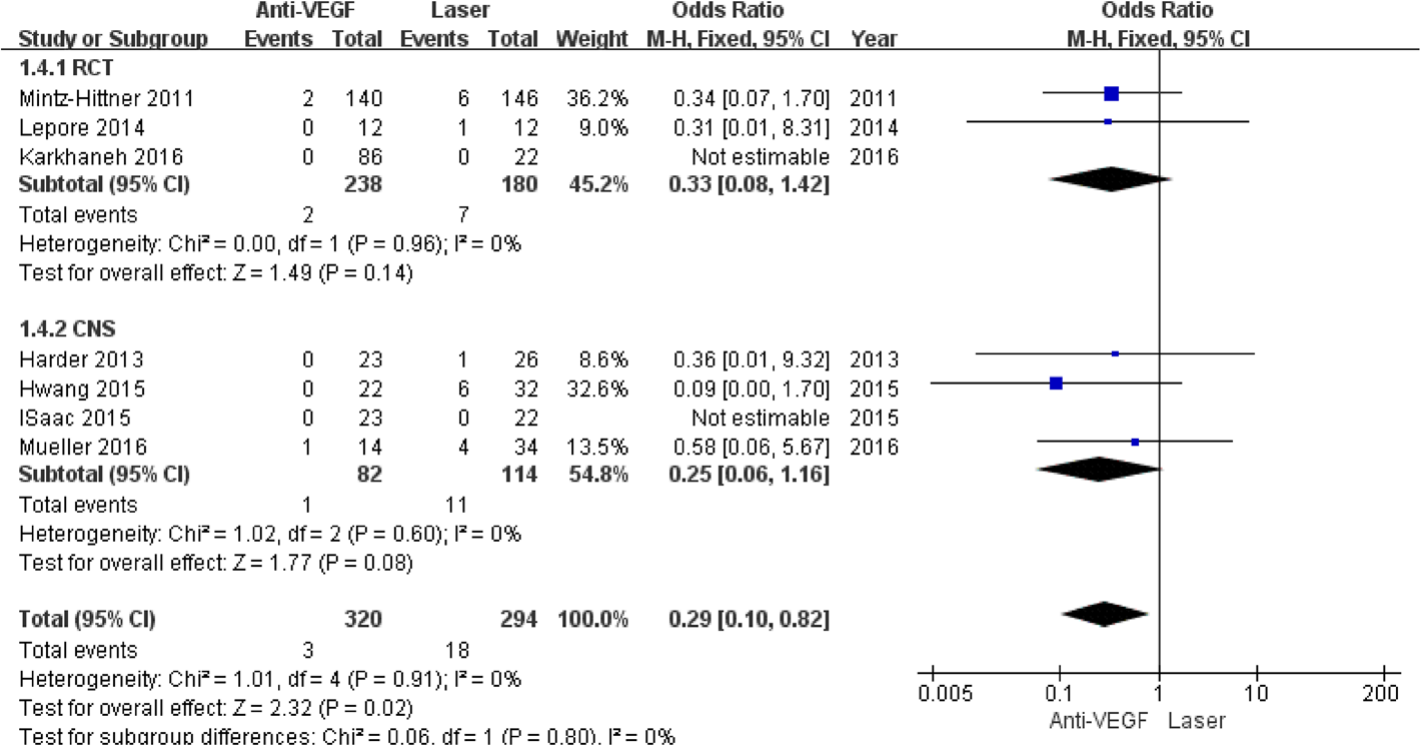
Forest plots depicting complication incidence reported in the included studies. ORs are shown with 95% CIs

**Fig. 7:**
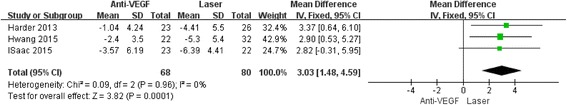
Forest plots depicting spherical equivalent reported in the included studies. WMDs are shown with 95% CIs

The eye complication incidence was significantly decreased in anti-VEGF (OR 0.29; 95% CI 0.10 to 0.82; *P* = 0.02) compared to laser with low heterogeneity in the results (I^2^ = 0%; *P* = 0.91) (Fig. 6). However, significance was not obvious for the eye complication incidence when the analyses were performed in each subgroup separately (RCT: OR 0.33, 95% CI 0.08 to 1.42, *P* = 0.14; CNS: OR 0.25, 95% CI 0.06 to 1.16, *P* = 0.08). In addition, myopia was also significantly decreased in anti-VEGF (WMD 3.03D; 95% CI 1.48 to 4.59; *P* = 0.0001) with low heterogeneity (I^2^ = 0%; *P* = 0.96) (Fig. 7).

## Discussion

The meta-analysis of 4 RCTs and 6 CNSs included 1158 type-1 and threshold ROP patients and compared the efficacy of anti-VEGF and laser treatment; the results showed that laser treatment was efficacious, with a significantly reduced retreatment incidence. However, the anti-VEGF treatment was safer, with a relatively reduced complication incidence and less myopia. No significant difference in the recurrence incidence or time between treatment and retreatment was found.

However, with no significant difference, the anti-VEGF and laser treatments had a similar recurrence incidence of 0% to 21.4% and 0% to 21.9%, respectively, which may show that both treatments were efficacious. A retrospective case series that demonstrated the recurrence of type 1 ROP after intravitreal bevacizumab (IVB) monotherapy was recently performed by Mintz-Hittner et al. [27]. IVB monotherapy was considered to be efficacious with a recurrence incidence of 7.2% (34/471) in the study. owever, Mintz-Hittner also suggested that the recurrence incidence may actually be higher with the anti-VEGF treatment. Consequently, frequent follow-up is more necessary with anti-VEGF to ensure timely retreatment.

High heterogeneity was found when the analysis was performed in terms of recurrence incidence. The different definitions of recurrence in the studies examined here may have contributed to the heterogeneity. Recurrent neovascularization, recurrent plus disease, and progression of traction were defined as recurrence in some studies, while termination of retinal vascularization and development of a demarcation line were included in the definition by others [9, 11].

Moreover, spontaneous regression occurred in some recurrent cases, thus requiring no retreatment. Therefore, compared to the recurrence incidence, the retreatment incidence is more meaningful for assessing efficacy in this meta-analysis. Laser treatment had a significantly reduced retreatment incidence when the retreatment incidence was examined. Changes in the levels of VEGF may account for the increased retreatment incidence in anti-VEGF intravitreal administration. The anti-VEGF antibody, which is available immediately after administration, decreases the levels of VEGF in the vitreous. When the levels of the anti-VEGF antibody in the vitreous gradually reduce and do not reach the effective concentration, increased levels of VEGF contribute to the development of neovascularization and progression. A similar notion was emphasized in Lorenz’s study [28]. Moreover, Xiang et al. [29] suggested that a compensatory mechanism existed in vascular growth factors in ROP. Other factors were upregulated when VEGF was expressed at a low level. Such a phenomenon may partially explain why anti-VEGF has higher a retreatment incidence in ROP. Another explanation for the superiority of laser treatment may be the great expertise of the surgeons in applying the laser. All surgeons of the included studies were senior surgeons who were experienced in laser treatment. Inexperienced surgeons often leave some of the area untreated, called the skipped area. The skipped area increases the risk of recurrence and requires retreatment. In addition, the quality and quantity of laser burns are crucial in the laser treatment. The explanation is consistent with the findings by Karkhaneh et al. and Kuo et al. [25, 30].

No significant difference was found in the time between treatment and retreatment in both groups. Because the recurrence time was not documented in most studies, the retreatment time was applied. However, the term was documented in only 3 studies, and the sample size was small. The result needs to be confirmed by further research. In our meta-analysis, the longest retreatment time was 17 weeks (PMA not more than 57 weeks), which means the recurrence time may be even shorter. The mean follow-up periods in all studies were variable, but they were not less than 24 weeks (PMA not more than 64 weeks). For anti-VEGF-treated eyes in particular, the follow-up period should be longer in case of recurrence. Consistently, a postmenstrual age (PMA) of 54 weeks was applied as the primary end-point for recurrence in the BEAT-ROP study [13]. A mean PMA of 70 weeks during the follow-up period was recommended in later studies because of late recurrence at 69 weeks PMA after IVB treatment [31, 32]. Recently, Gunay et al. [11] reported that no further recurrence was observed at PMAs of 62.90 weeks and 69.18 weeks in intravitreal ranibizumab (IVR)-treated and IVB-treated patients, respectively. A longer follow-up period (approximately PMA 70 weeks) may be suitable for the monitoring of recurrence when anti-VEGF is performed.

Safety is of great importance in the treatment options. In our analysis of complication incidence and myopia, the total sample size may be relatively small. We cannot draw precise and perfect conclusion only by these studies, but most of the included studies respectively did implied that a relatively reduced complication incidence and less myopia were found with the anti-VEGF treatment. Although laser has been considered to be the gold standard of ROP treatment in the past few decades, its disadvantages cannot be neglected. Laser burns destroy the full thickness of the peripheral retina, which makes it impossible for the retina to fully vascularize and differentiate. In addition, loss of the visual field, high myopia and cataracts are more common in laser-treated eyes. Supportive discussions can be found in several studies [8, 16, 33]. Specifically for high myopia, laser has been regarded as a risk factor, while anti-VEGF has been regarded as a protective factor [11, 15]. Many specialists highlighted the need of long-term follow-up, especially in laser-treated patients [34, 35]. However, Kuo et al. [30] found no significant difference in the myopic status of eyes that were treated with either anti-VEGF (27 eyes) or laser (26 eyes). The small sample size and short follow-up time may account for the result. A long and frequent follow-up for the refractive status is of great importance. The mechanism of myopia associated with laser in ROP patients is not well understood. A preserved peripheral retina in the anti-VEGF-treated eyes has been proposed to contribute to a normal emmetropization process [36]. Laser treatment has been suggested to be related to the inhibition of emmetropization and anterior segment development caused by the destroyed peripheral retina [37, 38].Abnormalities of anterior segment such as greater corneal curvature, shallow anterior chamber, long axial length and high lens power have been suggested [39, 40]. High crystalline lens power is suggested to be the predominant factor in some studies [41, 34].

Considering the side effect of laser treatment and the “simple” method of anti-VEGF intravitreal administration, experienced ophthalmologists tend to frequently apply anti-VEGF to type-1 and threshold ROP patients. The method requires only several minutes for the injection under intravenous sedation or topical anaesthesia. However, concerns for possible ocular and systemic side-effects remain in anti-VEGF intravitreal administration. One of the most serious eye complications is endophthalmitis, which is rare but devastating. In addition, according to Lepore’s study, significant vascular and macular abnormalities have been documented at the periphery or the posterior pole in anti-VEGF-treated eyes by fluorescein angiography (FA) 9 months after treatment. Lesions were not observed in most of the laser-treated eyes [24]. When rabbits were injected with 1.25 mg of IVB, the maximum serum concentration (MSC) reached 3.3 mg/ml 8 days after the injection [42]. The MSC in rabbits was consistent with that of 32-week-old premature infants [43], which suggested that a high concentration should be the focus regarding the systemic side-effects when serum concentration is considered. The assessment of neurodevelopmental outcomes after anti-VEGF has been performed in several studies [44, 45]. Although no significant neurodevelopmental impact was shown in some studies, neurodevelopmental delays were demonstrated in Morin’s study [46]. The evidence is not convincing enough, and the long-term effect remains unclear. A large-scale prospective RCT is needed to clarify the real impact of anti-VEGF on the neurodevelopment of ROP patients.

The meta-analysis has some limitations that should be acknowledged. First, among the 10 included studies, only four were RCTs. The statistical power to detect a difference was limited because some studies had a small sample size. Second, data such as the spherical equivalent were not recorded in some included studies. Only 3 eligible studies were retrieved with the terms “Spherical Equivalent in Last Follow-up” and “time between treatment and retreatment”. Moreover, the recurrence incidence or retreatment incidence was not reported in several studies, which might have an influence on the analysis. Third, heterogeneity arose between the two groups when the recurrence incidence and time between treatment and retreatment were compared. High heterogeneity probably affected the analysis outcomes. Even though a random-effects model was used, the effect of heterogeneity could be reduced but not abolished.

The heterogeneity of the studies could be mainly attributed to the following. In our meta-analysis, some studies conducted comparisons using a subtype of certain stages or zones, while others performed comparisons of all type-1 and threshold ROP patients. The stage and zone in ROP may be a related factor of the treatment efficacy. Consistently, in some studies, significant differences were shown in the efficacy of anti-VEGF between zones 1 and zone 2 in type-1 and threshold ROP. Gotz-Wieckowska et al. [47] reported that good anatomical results were achieved with laser in zone-2 and zone-3 ROP patients, compared to zone-1 patients. In addition, the type of anti-VEGF may also exert an influence on the efficacy. In some studies [11, 48], IVR-treated eyes had earlier and more frequent recurrences compared to IVB-treated ones. In contrast, Chen et al. [49] reported no significant difference in recurrence between IVB and IVR. Thus, research should be conducted regarding the above factors in the future. Lastly, the definition of recurrence, indicated above, is crucial to heterogeneity.

## Conclusion

This meta-analysis indicates that laser treatment may be more efficacious than anti-VEGF treatment regarding retreatment incidence. However, the results of this meta-analysis also suggest laser may cause more eye complications and higher myopia, which is related to further development of children’s visual function. Because anti-VEGF intravitreal administration is frequently applied, further assessment of the efficacy and safety between anti-VEGF and laser treatment should be performed. Large-scale prospective RCTs are needed to update the findings of this analysis.

## Additional files

Additional file 1: Electronic search strategy record. (DOCX 12 kb)

Additional file 2: PRISMA checklist. (DOCX 18 kb)

## Abbreviations

BEAT-ROP: Bevacizumab eliminates the angiogenic threat of retinopathy of prematurity
CNS: Comparative non-randomized studies
IVB: Intravitreal bevacizumab
IVR: Intravitreal ranibizumab
MSC: Maximum serum concentration
OR: Odds ratio
PMA: Postmenstrual age
PRISMA: Preferred reporting items for systematic reviews and meta-analyses
RCT: Randomized controlled trial
ROP: Retinopathy of prematurity
VEGF: Vascular endothelial growth factor
WMD: Weighted mean difference
