# Supplementary material for: Comparison of efficacy between anti-vascular endothelial growth factor (VEGF) and laser treatment in Type-1 and threshold retinopathy of prematurity (ROP)
Source: BMC Ophthalmol. 2018 Jan 30;18:19. doi: 10.1186/s12886-018-0685-6 (PMC5789737; doi:10.1186/s12886-018-0685-6)
Supplement: Supplementary file 1 — Electronic search strategy record. (DOCX 12 kb) [file 12886_2018_685_MOESM1_ESM.docx]

**S1 Text. Electronic search strategy record**

*Date*: 31 Mar 2017

*Database*：Embase，Pubmed

*Platform*: <http://www.embase.com/#advancedSearch/default>

<https://www.ncbi.nlm.nih.gov/pubmed/advanced>

*Retrieval formula*：

("ROP"[Supplementary Concept] OR "ROP"[All Fields] OR "rop"[All Fields]) OR ("retinopathy of prematurity"[MeSH Terms] OR ("retinopathy"[All Fields] AND "prematurity"[All Fields]) OR "retinopathy of prematurity"[All Fields]) ("1990/01/01"[PDAT] : "2017/03/31"[PDAT])

*Date limits*: 1 Jan 1990 to 31 Mar 2017

No any *other limits*
